# Cranial Tributary Ablation of the Saphenofemoral Junction during Laser Crossectomy of the Great Saphenous Vein

**DOI:** 10.3400/avd.oa.21-00066

**Published:** 2021-12-25

**Authors:** Tsuyoshi Shimizu, Yoshio Kasuga, Takeshi Shimizu

**Affiliations:** 1 Department of Cardiovascular Surgery, Nagano Matsushiro General Hospital, Nagano, Nagano, Japan; 2 Cosmos Nagano Clinic, Nagano, Nagano, Japan; 3 Department of Surgery, Nagano Matsushiro General Hospital, Nagano, Nagano, Japan

**Keywords:** varicose veins, endovascular procedures, catheter ablation, endovenous laser ablation, recurrent varicose veins

## Abstract

**Objectives:** Anterior accessory saphenous vein (AASV) insufficiency is one of the most common causes of recurrent varicose veins after endovenous thermal ablation (EVTA) for great saphenous vein (GSV) insufficiency. The purpose of this study was to evaluate the efficacy and safety of cranial tributary ablation (CTA) during laser crossectomy (LC) of the GSV.

**Methods:** We reviewed 182 limbs in 171 patients undergoing EVTA aiming for LC with a 1470-nm diode laser. In the CTA group, either the superficial circumflex iliac vein or the superficial epigastric vein was directly ablated during LC. The result was compared between the CTA (n=63) and control (n=119) groups using follow-up duplex ultrasound performed for 6 months after EVTA.

**Results:** Initial success rate of CTA was 69%. The AASV occlusion rate (90% vs. 63%, p<0.001) and the flush GSV occlusion rate (68% vs. 30%, p<0.001) at 6 months were better in the CTA group. No major adverse events were observed.

**Conclusion:** CTA during LC of the GSV is a safe and effective approach to achieve better flush or AASV occlusion rates after EVTA. It is occasionally technically demanding but can be a feasible option. Further investigation is needed to confirm our results.

## Introduction

Long-term results of endovenous thermal ablation (EVTA) for great saphenous vein (GSV) incompetence are still controversial^1)^; however, neoreflux in incompetent tributaries, such as the anterior accessory saphenous vein (AASV), is one of the most common causes of recurrence after EVTA.^2,3)^ On the other hand, this type of recurrence is less,^2,4)^ and neovascularization^5)^ is more common after high ligation and stripping because all saphenofemoral junction (SFJ) tributaries are generally ligated (flush ligation) at the time of surgery. Therefore, flush occlusion of the SFJ or the proximal GSV after EVTA can be associated with better long-term results without increasing the risk of neovascularization. Laser crossectomy (LC), in other words flush ablation or high ablation, is a promising approach; however, flush occlusion or AASV occlusion rates after LC are not always satisfactory. AASV flow was occasionally restored after flush occlusion of the GSV. Flow restoration or recanalization in the AASV developed either subsequent to or simultaneously with flow restoration in the cranial tributaries, such as the superficial circumflex iliac vein (SCI) or the superficial epigastric vein (SEV). Therefore, we believe that cranial tributary ablation (CTA) of the SFJ during LC could be associated with better flush occlusion rates or better AASV occlusion rates. The purpose of this study is to investigate the efficacy of this new approach.

## Methods

### Study design

We retrospectively analyzed 182 limbs in 171 patients undergoing EVTA aiming for LC of the GSV with a 1470-nm diode laser (ELVeS Radial 2 Ring™, Biolitec GmbH, Wiesbaden, Germany) since Jan 2017. Patients who underwent direct AASV ablation were excluded. Patients were divided into two groups based on the treatment performed on their limbs: the CTA group (63 limbs), which underwent CTA during LC, and the control group (119 limbs), which underwent LC alone. Patients in whom CTA was attempted but unsuccessful (28 limbs) were classified into the control group.

Follow-up examinations using duplex ultrasound were performed for 6 months after EVTA, and the results were compared between the groups.

### LC technique

**Figure 1:**
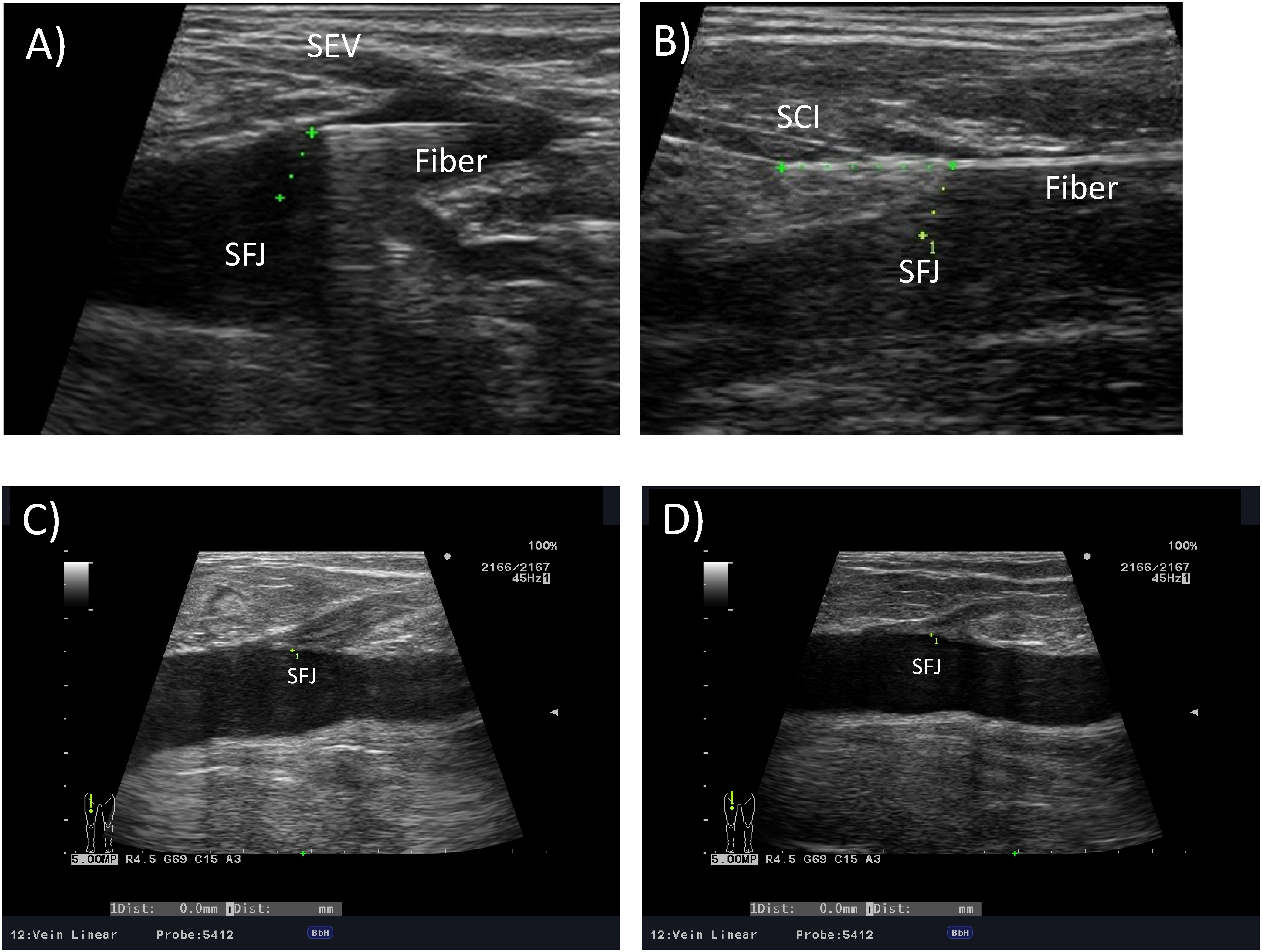
Fig. 1 Positioning of the fiber tip during endovenous thermal ablation and flush occlusion. (**A**) Duplex ultrasound shows the fiber tip positioned close to the saphenofemoral junction (SFJ) proximal to the superficial epigastric vein (SEV) for laser crossectomy. (**B**) Duplex ultrasound shows the fiber tip inserted into the superficial circumflex iliac vein (SCI) for cranial tributary ablation. (**C**) Duplex ultrasound shows flush occlusion of the great saphenous vein obstructed just at the SFJ 3 months after laser crossectomy with concomitant superficial circumflex iliac vein ablation. (**D**) The great saphenous vein remained occluded at the SFJ 6 months after endovenous ablation (**C** and **D** are images from the same patient).

The target GSV and the SFJ, including its tributary distributions, were assessed using duplex ultrasound before EVTA. The fiber was delivered into the GSV and advanced to the SFJ. Ablation was commenced at about 5 mm from the SFJ (**Fig. 1A**). High energy (300–500 J/cm) was applied to the proximal portion (1–2 cm) of the GSV with the femoral vein (FV) compressed in the Trendelenburg position.

### CTA technique

The fiber was delivered into the GSV and advanced up to the SFJ. Under ultrasound guidance, the fiber tip was introduced into the SCI or the SEV. To visualize the SEV, the GSV, and the fiber in the same longitudinal plane, the probe was tilted laterally. To visualize the SCI, the probe was tilted medially, and the fiber was directed laterally. The operator held the probe with the hand and manipulated the fiber with the other hand from the body surface to direct the fiber tip toward the orifice of the SCI/SEV. The assistant advanced the fiber approximately 10 mm into the SEV/SCI (**Fig. 1B**). The time for fiber cannulation into the SEV/SCI was limited within approximately 5 min. After tumescent local anesthesia, the tributary was directly ablated with 50–100 J. Subsequently, the fiber tip was taken out to the proximal GSV and advanced toward the SFJ, and the proximal GSV was ablated close to (5 mm) the SFJ using the crossectomy technique as described previously. Initial success of CTA was defined as the target tributary being ablated under successful fiber cannulation into the SCI/SEV >5 mm from the confluence during LC.

### Follow-up

Follow-up examinations using duplex ultrasound were performed 1 day, 1 week, 1 month, and 3 and 6 months after EVTA. Patency or occlusion of the SFJ tributaries was confirmed using color doppler ultrasound. Patent tributaries were defined as follows: the confluence of the tributaries and the GSV were not completely occluded, and venous blood flow in the tributaries returned to the FV through the proximal GSV. The flush occlusion of the proximal GSV was defined as the GSV occlusion just or near the SFJ without any flow from the SFJ tributaries to the SFJ (**Figs. 1C** and **1D**).

### Ethical approval

Written informed consent was obtained from all patients. The ethics committee in our institute approved this study (reference number 274).

### Statistical analysis

Continuous variables were reported as the mean with standard deviation for parametric distributions and compared using Student’s t test. Categorial data were compared using the Chi-square or Fisher Exact test. Statistical significance was assumed at p<0.05.

## Results

CTA was attempted in 91 limbs in the latter half of the study period but failed in 28 limbs due to failed cannulation. The initial success rate of CTA was 69%. There were no significant differences in baseline characteristics, including SFJ tributary distributions between the groups (**Table 1**).

**Table 1:** Table 1 Baseline characteristics

	CTA group		Control group		P value
No. of limbs	63		119		
Age (year old)	62	+/− 13	64	+/− 12	0.295
Male	27	(43%)	45	(38%)	0.508
Body mass index	23.8	+/− 3.2	22.8	+/− 3.2	0.073
CEAP >3	11	(17%)	26	(22%)	0.484
Proximal GSV diameter (mm)	8.6	+/− 3.0	8.8	+/− 2.2	0.514
AASV distribution					
AASV draining to GSV	45	(71%)	80	(67%)	0.999
AASV draining to FV	0	(0%)	5	(4%)	0.166
AASV draining to SCI	7	(11%)	9	(8%)	0.421
Absent AASV	11	(17%)	25	(21%)	0.568

CTA: cranial tributary ablation; CEAP: The Clinical-Etiology-Anatomy-Pathophysiology Classification; GSV: great saphenous vein; AASV: anterior accessory saphenous vein; FV: femoral vein; SCI: superficial circumflex iliac vein

In the CTA group, the SCI was ablated in 34 limbs, the SEV was ablated in 26 limbs, and the common trunk of the SEV and the SCI in 3 limbs. The mean ablated tributary length was 9.9+/−3.9 mm, and the mean laser energy applied was 88+/−19 J. A slim fiber was used more frequently in the CTA group, but there were no significant differences in other operative factors and early postoperative results between the groups (**Table 2**). Endovenous heat-induced thrombosis (EHIT) class 3 (Kabnick classification)^6)^ was found in one limb in the control group, but none in the CTA group. No other adverse events were observed in both groups.

**Table 2:** Table 2 Operative factors and early postoperative results

	CTA group		Control group		P value
Operative factors					
No. of limbs	63		119		
Laser fiber					
Regular	43	(68%)	102	(86%)	0.005
Slim	20	(32%)	17	(14%)	0.005
Laser power (W)	8.4	+/− 1.6	8.6	+/− 1.3	0.349
Treated GSV length (cm)	35	+/− 10	33	+/− 10	0.378
LEED (J/cm)	93	+/− 15	90	+/− 21	0.213
Microphlebectomy	4.4	+/− 2.2	4.7	+/− 2.1	0.292
Tumescent local anesthesia (ml)	215	+/− 57	214	+/− 50	0.848
Operation time	48	+/− 11	46	+/− 12	0.306
Early postoperative results					
EHIT on 1 day after EVTA					
Class 2	7	(11%)	11	(9%)	0.688
Class 3	0	(0%)	1	(1%)	0.999
GSV occlusion at one week	63	(100%)	119	(100%)	0.999
Venous thromboembolism	0	(0%)	0	(0%)	0.999
Paresthesia	1	(2%)	0	(0%)	0.999
Bruise	25	(40%)	45	(38%)	0.805
Pain	31	(49%)	58	(49%)	0.952

CTA: cranial tributary ablation; GSV: great saphenous vein; LEED: liner endovenous energy density; EHIT: endovenous heat-induced thrombosis; EVTA: endovenous thermal ablation

**Figure 2:**
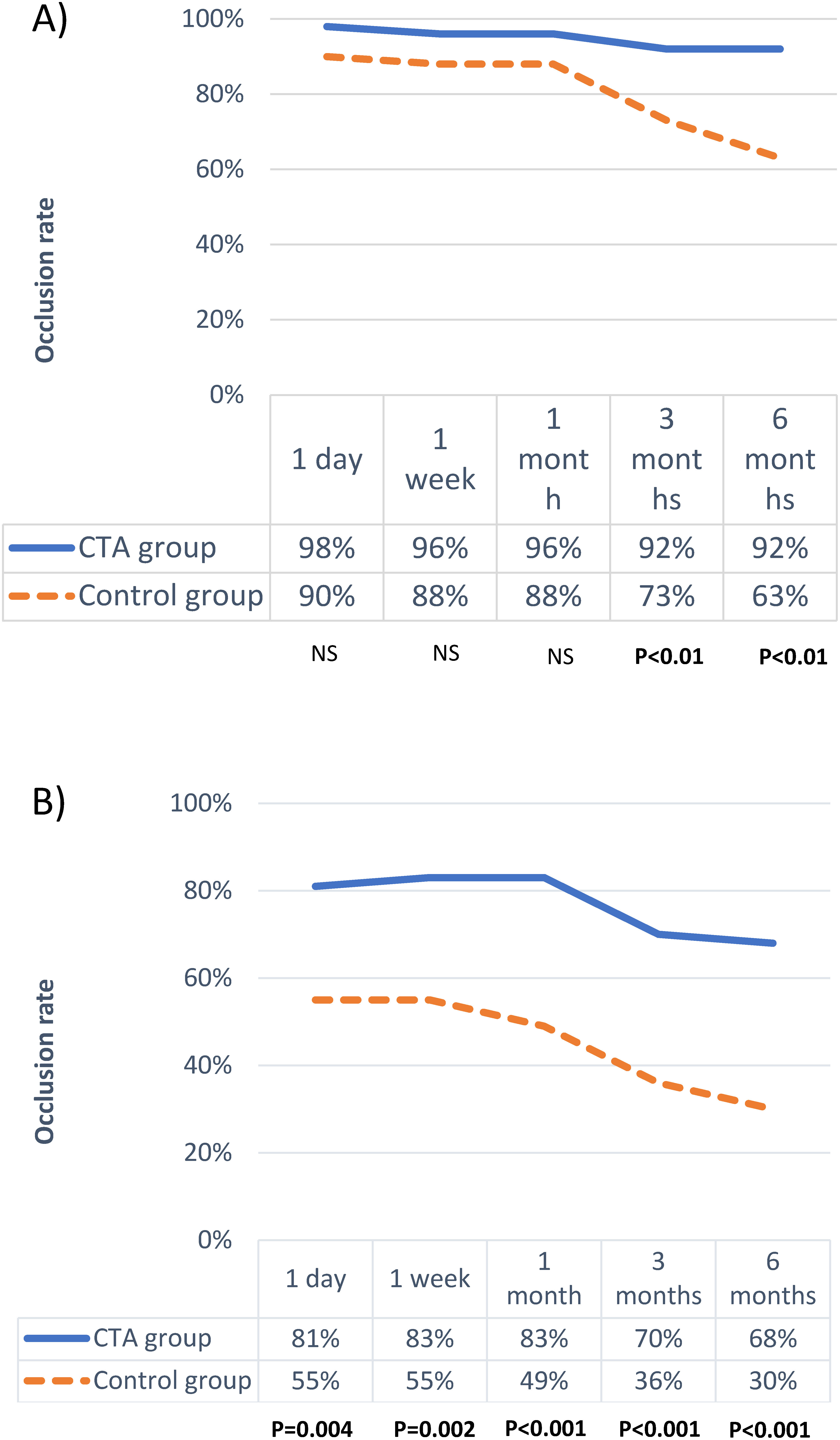
Fig. 2 Saphenofemoral junction tributary vein occlusion rate after endovenous thermal ablation for 6 months. (**A**) anterior accessory saphenous vein occlusion rate and (**B**) flush occlusion rate.

The AASV occlusion rates at 3 and 6 months were significantly better in the CTA group (**Fig. 2A**). In the control group, 33% of the occluded AASVs showed recanalization at 6 months compared with only 4% in the CTA group (p<0.01). Neoreflux of the patent or recanalized AASV was not seen in both groups. The flush occlusion rates were significantly higher in the CTA group during the 6-month follow-up period (**Fig. 2B**). The GSV occlusion rate was 100% in both groups for 6 months after EVTA.

## Discussion

In the CTA group, the occlusion rates of the AASV were better at 3 and 6 months after EVTA while the rates were comparable at one month after EVTA. These data suggest that CTA could predominantly reduce recanalization of the AASV. Muhlberger et al.^7)^ reported that the AASV and the SEV often joined the GSV at 1–2 cm from the SFJ. When ablation is commenced at a point 1–2 cm distal from the SFJ,^8)^ the confluence of the AASV and the GSV is often not ablated at the time of EVTA; consequently, communication between the AASV and the SFJ is preserved after EVTA. Thus, starting ablation closer to the SFJ may be associated with better AASV occlusion rates.

The bare-tip fiber releases its energy in a forward direction, whereas the energy from the radial fiber is emitted radially in a 360°-manner from two prisms at the fiber tip. Therefore, compared with a bare-tip fiber, a radial fiber could be placed closer to the junction. Shimizu^9)^ suggested that the occlusion rate of the AASV was very low after EVTA (980-nm laser) because a patent AASV was observed in 70% of the patients at 2 years after EVTA in his 3-year follow-up study. We often found that the GSV that had been initially occluded proximal to the AASV reopened at the confluence of the AASV several months after EVTA (980-nm laser).

In our study using the 1470-nm laser, the AASV occlusion rate at 6 months after EVTA was 63% in the control group. Compared with EVTA (980-nm laser), the occlusion rate of the AASV was better when the 1470-nm laser was used without CTA. Although one of the reasons was due to fiber position, the others included laser profile, such as wavelength. The laser energy level applied to the proximal GSV could have been one of the contributing factors. We applied 300–500 J to the proximal portion of the GSV to secure flush occlusion or AASV occlusion.

**Figure 3:**
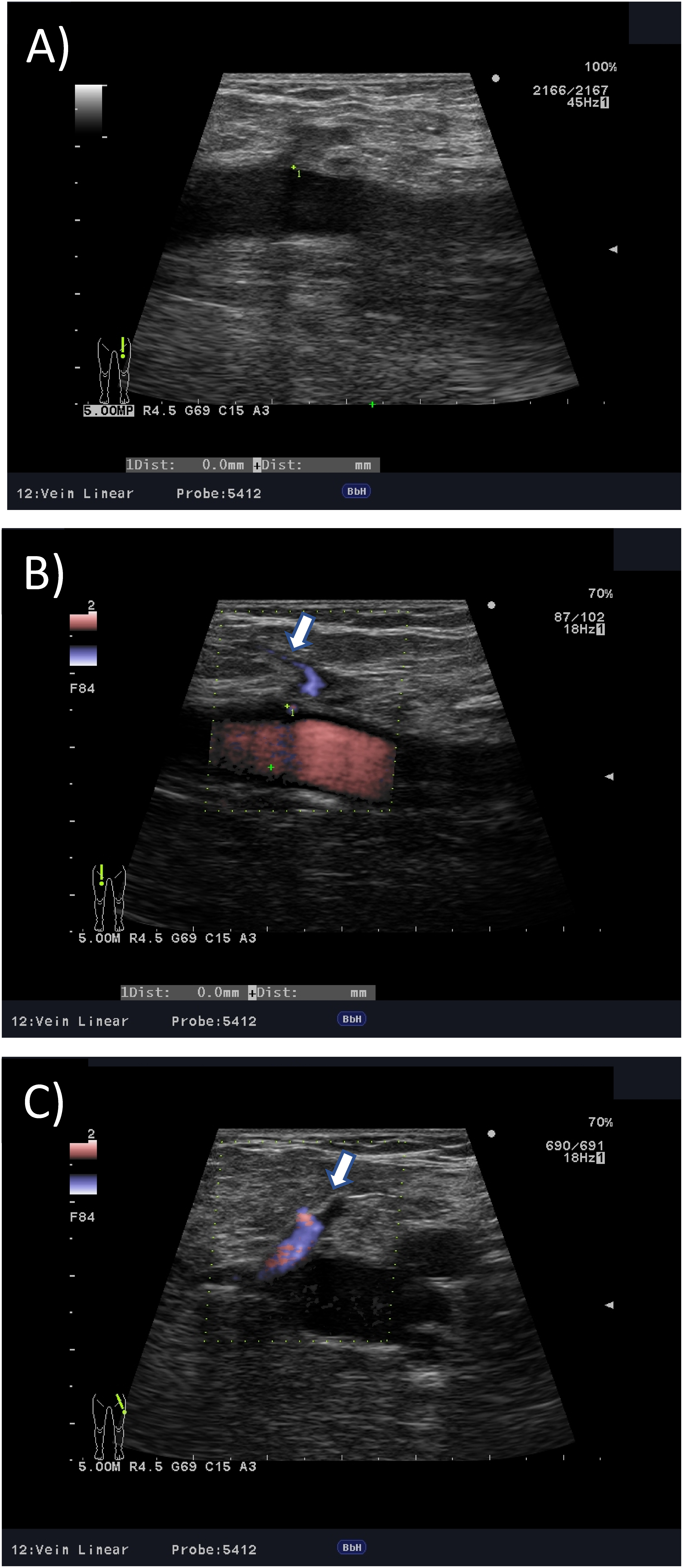
Fig. 3 One process leading to anterior accessory saphenous vein recanalization after laser crossectomy. Duplex ultrasound showed flush occlusion of the great saphenous vein at one month (**A**), superficial epigastric vein recanalization (arrow) at 3 months (**B**), and anterior accessory saphenous vein recanalization (arrow) at 6 months (**C**) after laser crossectomy.

In the control group, 33% of the occluded AASV showed recanalization at 6 months. In those cases, the AASV sometimes joined the GSV adjacent to the SFJ along with other tributaries, such as the SCI or the SEV. We encountered a patient whose proximal GSV was occluded at the SFJ completely at 1 month, but SEV flow was restored at 3 months, and AASV flow was subsequently restored at 6 months after EVTA (**Fig. 3**). Recanalization of the cranial branch that drains the GSV close to the junction might induce recanalization of the distant tributary, such as the AASV. This may be one process leading to AASV recanalization after flush occlusion of the ablated GSV. Therefore, reduction of the number of patent tributaries near the junction in the early postoperative period can be another contributing factor for further AASV occlusion. In this study, flush occlusion rate was also better in the CTA group; however, flush occlusion is not a goal but a means to secure AASV occlusion.

Direct ablation of the AASV is more effective in preventing AASV insufficiency following EVTA; however, it is almost anatomically impossible to advance the laser fiber tip into the AASV from the GSV at the time of usual GSV ablation. On the other hand, we sometimes experience an accidental insertion of the fiber tip into the SEV. Among the SFJ tributaries, the SEV or the SCI is more accessible intraluminally from the GSV because of anatomical characteristics. For CTA, either the SEV or the SCI should be selected because cannulation of the fiber into the SEV/SCI after tumescent local anesthesia is technically difficult. The SEV is generally more accessible compared with the SCI. The SCI and the AASV drains into the GSV anteriorly and laterally. The SCI generally drains closer to the SFJ.^7)^ The SCI and the AASV often drains into the GSV close to each other. Moreover, the AASV sometimes drains into the SCI itself.^10)^ Therefore, the SCI was more frequently selected for CTA. Large tributaries were also selected for CTA.

This technique cannot always be recommended to patients with GSV incompetence. Good indications of this technique include the following: ablation of the SEV/SCI, which drains into the GSV close to (the distance from the SFJ to the SEV/SCI <10 mm, especially <5 mm) the SFJ with the AASV or posterior accessory saphenous vein (PASV), ablation of the SCI into which the AASV drains, ablation of large or dilated SCI/SEVs, and ablation of the SEV into which the PASV drains. The patent PASV also causes recurrence of varicose veins after EVTA.

EHIT could be one of the possible complications after this technique because some investigators hypothesized that the SEV might have some role in preventing EHIT.^11)^ In this study, the incidence of EHIT class 2 or over in the CTA group was comparable to that in the control group. CTA itself did not increase the risk of EHIT. It is controversial whether the distance from the SFJ to the fiber tip is associated with an increase in the incidence of EHIT or not.^12,13)^ In our study, the incidence of EHIT class 2 in both groups could have been higher compared with previous reports.^14,15)^ LC might increase the risk of EHIT; however, EHIT class 2 is generally not treated with anticoagulants in Japan.^15)^ Moreover, EHIT do not typically develop a symptomatic venous thromboembolism after EVTA.^14,16)^ Neither critical EHIT nor venous thromboembolism had developed among the patients in our study.

Neovascularization is possible pathophysiological change after CTA. Mariani et al.^17)^ suggested that it was important to maintain the venous flow of the tributaries coming from the abdominal wall to the common FV because selective high ligation that preserved the SEV or SCI might decrease the incidence of neovascularization and recurrent varicose veins in the operated groin after high ligation and stripping (HLS). However, their study was not a control trial. The incidence of neovascularization after HLS have been varied^18–20)^ and could be reduced by some other surgical techniques, such as suture of the GSV stump^19,21)^ or patch saphenoplasty.^22)^ The patients in this study are still in the follow-up observation period for 2 years after EVTA. Neovascularization has been rare even in patients with flush occlusion of the GSV 1 or 2 years after EVTA. We believe that LC will not induce neovascularization. Long-term follow-up is needed to confirm our results.

External pudendal artery injury, stuck fibers, or arteriovenous fistula formation^23)^ around the SFJ are other possible complications with this technique. However, we have not experienced these complications so far by applying appropriate laser energy (50–150 J) for SEV/SCI ablation and adequate volumes of tumescent anesthesia. Higher energy levels may be associated with these complications.

This study has some limitations. The CTA technique cannot be performed by one operator. A scrubbing or circulating assistant is required to insert the fiber into the SEV/SCI; however, CTA can be performed at almost no additional cost and no extra charge. A potential learning curve exists to decrease the procedure time and increase the success rate of this technique. The initial success rate of CTA was 69% in this study. The slim fiber may be more flexible in accessing the SEV/SCI, but we could not demonstrate its superiority. There are also limitations in manipulating the fiber from the skin surface. This study was not a randomized control study. The total number of limbs, especially the ones treated with a slim fiber, was small.

## Conclusion

CTA (SCI/SEV) of the SFJ during LC of the GSV is a safe and effective approach to achieve better AASV occlusion rates after EVTA. It is occasionally technically demanding but can be a feasible option. Further investigation is needed to confirm the long-term efficacy of this technique.

## Additional Note

We presented an overview of our manuscript at the International Session of the 40th Annual Scientific Meeting of the Japanese Society of Phlebology on September 17, 2020 on the web.
